# Impacts of breast cancer and chemotherapy on gut microbiome, cognitive functioning, and mood relative to healthy controls

**DOI:** 10.1038/s41598-022-23793-7

**Published:** 2022-11-15

**Authors:** Emily Bilenduke, John D. Sterrett, Krista W. Ranby, Virginia F. Borges, Jim Grigsby, Alaina L. Carr, Kristin Kilbourn, Christopher A. Lowry

**Affiliations:** 1 Department of Psychology, University of Colorado Denver, Denver, CO USA; 2 Department of Integrative Physiology and Center for Microbial Exploration, University of Colorado Boulder, Boulder, CO USA; 3 Interdisciplinary Quantitative Biology, University of Colorado Boulder, Boulder, CO USA; 4 Department of Medicine, University of Colorado School of Medicine, Aurora, CO USA; 5 Department of Oncology, Georgetown Lombardi Comprehensive Cancer Center, Georgetown University, Washington, DC USA; 6 Department of Psychology and Neuroscience and Center for Neuroscience, University of Colorado Boulder, Boulder, CO USA; 7 Department of Physical Medicine and Rehabilitation and Center for Neuroscience, University of Colorado Anschutz Medical Campus, Aurora, CO USA; 8 Veterans Health Administration, Rocky Mountain Mental Illness Research Education and Clinical Center (MIRECC), Rocky Mountain Regional Veterans Affairs Medical Center (RMRVAMC), Aurora, CO USA; 9 Military and Veteran Microbiome: Consortium for Research and Education (MVM-CoRE), Aurora, CO USA

**Keywords:** Breast cancer, Microbiome, Psychology

## Abstract

Women diagnosed with breast cancer undergoing chemotherapy experience cognitive impairment, symptoms of anxiety and depression, and physical side effects including disruption in the diversity and community composition of the gut microbiome. To date, there is limited research exploring the associations among these specific challenges. The present cross-sectional study explored the associations of self-reported cognitive functioning, depression, and anxiety symptoms, and gut microbiome diversity and community composition in women who were diagnosed with and undergoing chemotherapy treatment for breast cancer (BC) compared to cancer-free healthy controls (HC). The BC group displayed higher rates of cognitive dysfunction (*p* < 0.001) and depressive symptoms (*p* < 0.05) relative to HC. There was a significant difference in microbiome community composition between BC and HC, particularly characterized by a decreased relative abundance of the mucin-degrading genus *Akkermansia* in BC compared to HC (*p* < 0.05). Association models identified significant associations among group, cognitive, depression, and microbiome variables (*p* < 0.001). Overall, the study identified that BC participants experienced significant differences in self-reported cognitive functioning, self-reported depression symptoms, microbiome community composition, and mucin-degrading bacteria of the gut-mucosal barrier, relative to HC. The present study is consistent with the hypothesis that gut microbiome community composition impacts a woman’s experience with breast cancer and treatment suggesting that microbiome-based interventions have potential for improving quality of life outcomes in individuals with breast cancer.

## Introduction

Breast cancer is the second most common cancer to occur in women. It is estimated that one in eight women in the United States will develop breast cancer during their lifetime and 268,600 new cases of invasive breast cancer are diagnosed in women every year in the United States. Treatment advances and early detection screenings have increased survival rates from 74.8% 5-year survival rate in 1975 to a 91.1% 5-year survival rate in 2014^1^. It is estimated that 82.4% of individuals diagnosed with breast cancer will receive pre-adjuvant or adjuvant chemotherapy treatment^1^. While chemotherapy has tremendously impacted survival rates, it also comes with significant side effects that impact a person’s physical health, mental health, social well-being, and quality of life.

Chemotherapy aims to reduce cancer cell growth by targeting rapidly dividing cells; however this means that other cells including hematopoietic stem cells, hair cells, cells of the central nervous system (CNS), and cells of the mucous membranes within the mouth, throat, and digestive system are targeted by the treatment^2^. The resulting increased systemic inflammation from chemotherapy’s action can lead to significant side effects. For instance, transcription of nuclear factor kappa B (NFκB), a master regulator of inflammatory signaling cascades throughout the body [including tumor necrosis factor alpha, interleukin (IL)-1β, IL-6, and IL-8] is upregulated by common chemotherapy drugs^3^. Notably, this increased inflammation is not limited to the periphery: it also occurs in the CNS through multiple mechanisms including through actions on the adaptive and innate immune systems, such as increased production of chemokines and cytokines^3^. Such inflammation increases risk of significant side effects such as impaired cardiovascular function, altered hormone concentrations, and sensitized pain circuits. Most notably for this study, though, the neuroinflammation impacts mood and cognitive function^4^, and these side effects can significantly decrease a person’s quality of life.

Cognitive impairment, depression and anxiety symptoms are common in individuals undergoing chemotherapy. Evidence suggests that chemotherapy impairs cognition, including impairment of short-term memory^5^, attention and concentration^6^, long-term memory, processing speed, and overall executive functioning^7^. Longitudinal neuropsychological assessment of cancer patients during treatment determined that up to 75% of patients report changes in memory, executive functioning, and attention/concentration as assessed through self-report measures^8^. In addition, breast cancer patients report more depression and anxiety symptoms than members of the general population^4,9–12^, especially women with breast cancer who are being treated with chemotherapy^4,11^. One of the hypothesized mechanisms explaining the increased rates of cognitive dysfunction and increases in depression and anxiety symptoms is increased gut permeability, increased endotoxin in circulation (as evidenced by increases in lipopolysaccharide binding protein, a biomarker of gut permeability), systemic inflammation and neuroinflammation^2,13,14^, suggesting that, outside of increased general stress, there exist physiological mechanisms that increase these mood and cognitive symptoms during chemotherapy.

Although the mechanisms underlying increased inflammation and neuroinflammation in response to chemotherapy, as well as individual variability in these responses, are not fully understood, evidence suggests that the gut microbiome may play an important role. The gut microbiome can modulate brain activity and behavior through the nervous, neuroendocrine, and immune systems^15,16^. However, both the gut microbiome and gut mucosa (at the interface of the host and microbiome) are disrupted by chemotherapy. Previous research has demonstrated significant changes in microbiome composition in persons receiving chemotherapy treatment, resulting in activation of the neuroimmune system and increased expression of proinflammatory cytokines^2,14,17^. Therefore, the disruption of the gut microbiome and mucosal barrier can increase stress responsiveness^18^, inflammation, and immune response^19^, including neuroinflammation.

Disruption of the diversity and community composition of the microbiome from a healthy state, i.e., dysbiosis, is associated with fatigue, cognitive problems such as a change in memory and executive functioning, and mood changes^15^ including depression^15,16,18–20^. Previous research establishes that women with breast cancer experience significant side effects as a result of chemotherapy treatment that impact their functioning and quality of life. Side effects include impairment of cognitive functioning, depression symptoms, anxiety symptoms, and disruption of the gut microbiome. However, there is limited research looking at the relationship between cognitive functioning, depression symptoms, anxiety symptoms, and the gut microbiome in women who were diagnosed with and undergoing chemotherapy treatment for breast cancer (BC); therefore, in the present study we explored associations among these variables in BC compared to healthy controls (HC).

## Results

Demographic data were collected and compared for the two groups (Table 1). Overall, the ages of participants ranged from 19 to 74 with an overall mean age of 46.7 years and 86% of the sample identified as White (non-Hispanic). A chi-square test of independence was conducted between participation status and the demographic variables. There were no significant differences in age, racial/ethnic group, education, relationship status, annual income, and area of living. There was a significant difference between the groups regarding having children *X*^*2*^ (1) = 6.2, *p* = 0.01, such that the BC group (*n* = 15; 71%) reported having children more than the HC group (*n* = 4; 29%).

**Table 1:** Self-report subject characteristics of women who were diagnosed with and undergoing chemotherapy treatment for breast cancer (BC) and healthy control (HC) groups, and all study subjects.

	BC	HC	All
	(*n* = 21)	(*n* = 14)	(*N* = 35)
**Age, years**			
Mean (± SD)	51.7 (10.8)	39.1 (15.8)	46.7 (14.4)
Range	30–67	19–74	19–74
**Sex, *n* (%)**			
Female	21 (100)	14 (100)	35 (100)
Male	0 (0)	0 (0)	0 (0)
**Race/ethnicity *n* (%)**			
White (not of Hispanic origin)	20 (95)	10 (71)	30 (86)
Hispanic	1 (5)	2 (14)	3 (9)
African American/Black	0 (0)	0 (0)	0 (0)
Asian/Pacific Islander	0 (0)	2 (14)	2 (6)
Multi-ethnic	0 (0)	0 (0)	0 (0)
**Education—years, *n* (%)**			
Less than high school—11	0 (0)	0 (0)	0 (0)
High school—12	2 (10)	0 (0)	2 (6)
College—13	1 (5)	1 (7)	2 (6)
College—14	1 (5)	0 (0)	1 (3)
College—15	1 (5)	1 (7)	2 (6)
College—16	7 (33)	3 (21)	12 (34)
Associate degree	4 (19)	0 (0)	2 (6)
Post-graduate—17	2 (10)	1 (7)	3 (8)
Post-graduate—18+	3 (14)	8 (57)	11 (31)
**Marital status *n* (%)**			
Married	11 (52)	7 (50)	18 (52)
Divorced	4 (19)	0 (0)	4 (11)
Committed relationship (partner opposite sex)	3 (14)	2 (14)	5 (14)
Single (never married)	3 (14)	5 (36)	8 (23)
**Children *n* (%)**			
Yes	15 (71)	4 (29)	19 (54)
No	6 (29)	10 (71)	16 (46)
**Annual income *n* (%)**			
$0–$25,000	1 (5)	1 (7)	2 (6)
$26,000–$50,000	2 (9)	4 (29)	6 (17)
$51,000–$75,000	3 (14)	1 (7)	4 (11)
$76,000–$100,000	6 (29)	2 (14)	8 (23)
$100,000+	9 (43)	6 (43)	15 (43)
**Area of living *n* (%)**			
Urban	10 (48)	5 (36)	15 (43)
Rural	4 (19)	2 (14)	6 (17)
Suburban	7 (33)	7 (50)	14 (40)

Data are displayed as *n* (%) or mean (± SD).

The BC group included participants with stage I through stage III breast cancer. Post-menopausal women represented 57% of the group and 7 different chemotherapy treatment combination/sequences were administered to BC participants. For the BC group, the average time between most recent chemotherapy treatment and self-report psychological questionnaire completion was 11 days. The average time between most recent chemotherapy treatment and completion of the stool sample was 13 days (Table 2).

**Table 2:** Subject characteristics for the women who were diagnosed with and undergoing chemotherapy treatment for breast cancer (BC).

Characteristics	Frequency (%)
**Cancer stage**	
I	4 (19)
II	9 (43)
III	4 (19)
Not identified/missing	4 (19)
**Menopause status**	
Post-menopausal	12 (57)
Pre-menopausal	9 (43)
**Chemotherapy drugs**	
Included as part of total treatment regimen	
Doxorubicin	8
Capecitabine	1
Carboplatin	10
Cyclophosphamide	10
Docetaxel	12
Paclitaxel	7
**Chemotherapy treatment combination/sequence**	
Treatment received up to enrollment date	
Doxorubicin, cyclophosphamide	2 (10)
Doxorubicin, cyclophosphamide, paclitaxel	5 (23)
Doxorubicin, cyclophosphamide, paclitaxel, capecitabine	1 (5)
Docetaxel, carboplatin, trastuzumab, pertuzumab	8 (38)
Docetaxel, carboplatin, trastuzumab	2 (10)
Docetaxel, cyclophosphamide	2 (10)
Paclitaxel	1 (5)
**Mean time**	**Days (± SD)**
Average time between most recent treatment and psychological questionnaires	11.4 (11.3)
Range of time between most recent treatment and psychological questionnaires	0–40
Average time between most recent treatment and stool samples	13 (14.4)
Range of time between most recent treatment and stool samples	0–42

Data are displayed as *n* (%), mean days (± SD).

### Psychosocial outcomes

**Figure 1:**
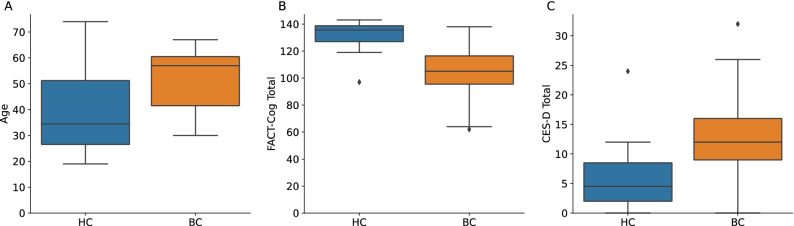
Boxplots depict (**A**) age, (**B**) Center for Epidemiological Studies Depression Scale (CES-D) total score, and (**C**) Functional Assessment of Cancer Therapy-Cognitive Function (FACT-Cog) total score across women who were diagnosed with and undergoing chemotherapy treatment for breast cancer (BC) and healthy controls (HC). Boxes represent the first and third quartiles, and horizontal lines within boxes represent median values. Whiskers represent non-outlier high and low values, and diamonds represent outliers as defined by having a value greater than 1.5 times the interquartile range (IQR) of the first or third quartile. In (**B**), lower FACT-Cog values in BC indicate more cognitive difficulties, and in (**C**), higher CES-D scores in BC indicate higher depressive symptoms.

A one-way analysis of variance (ANOVA) was conducted to examine the differences between The Functional Assessment of Cancer Therapy-Cognitive Function (FACT-Cog) scores for the two groups. The difference in FACT-Cog scores between BC and HC was statistically significant, *F*(1,34) = 21.7, *p* < 0.001, partial *ƞ*^2^ = 0.4 (Table 3; Fig. 1). The BC group had lower mean (± SD) FACT-Cog scores (103.9 ± 19.7) than HC (131.4 ± 12.1). The significantly lower scores of the BC group indicate greater reported cognitive difficulties than the HC group. Additionally, there were significant differences between the two groups in the following subscales: *perceived cognitive impairments*, *perceived cognitive abilities,* and *quality of life*. For the *perceived cognitive impairments* subscale, BC (55.1 ± 13.1) had statistically significant lower scores on *perceived cognitive impairments* scores than HC (69.1 ± 6.2), *F*(1,34) = 13.9, *p* = 0.001, partial *ƞ*^2^ = 0.3. For the *perceived cognitive abilities* subscale, BC (22.3 ± 6.1) had lower *perceived cognitive abilities* score than HC (31.4 ± 4.9), *F*(1,34) = 21.6, *p* < 0.001, partial *ƞ*^*2*^ = 0.4. For the *quality of life* subscale, BC (11.6 ± 3.7) had lower *quality of life* scores than HC (15.2 ± 1.6), *F*(1,34) = 11.8, *p* = 0.002, partial *ƞ*^*2*^ = 0.3. Finally, for the *comments from others* subscale, BC (1.1 ± 1.5) had statistically significant higher scores than HC (0.2 ± 0.4), *F*(1,34) = 4.1, *p* = 0.05, partial *ƞ*^2^ = 0.1 (Table 3).

**Table 3:** Analysis of Variance (ANOVA) between the women who were diagnosed with and undergoing chemotherapy treatment for breast cancer (BC) and healthy control (HC) groups on psychosocial measures of cognitive functioning, depression, anxiety, and perceived stress.

Variable	Mean (± SD)	Range	*F*	*p*	*ƞ*^2^
**FACT-Cog total**					
BC	103.9 (19.7)	62–138	21.7	0.001***	0.4
HC	131.4 (12.1)	97–143			
**Perceived cognitive impairments**					
BC	55.1 (13.1)	19–72	13.9	0.001***	0.3
HC	69.1 (6.2)	52–75			
**Perceived cognitive abilities**					
BC	22.3 (6.1)	12–35	21.6	0.001***	0.4
HC	31.4 (4.9)	19–36			
**Comments from others**					
BC	1.1 (1.5)	0–5	4.1	0.05*	0.1
HC	0.2 (0.4)	0–1			
**Quality of life**					
BC	11.6 (3.7)	1–16	11.8	0.002**	0.3
HC	15.2 (1.6)	10–16			
**CES-D**					
BC	11.9 (7.7)	0–32	5.4	0.03*	0.1
HC	6.1 (1.9)	0–24			
**PROMIS Bank v1.0–anxiety**					
BC	50.8 (7.7)	33–63	1.4	0.3	0.04
HC	47.8 (6.4)	33–59			
**PSS**					
BC	13.7 (4.9)	6–22	0.9	0.4	0.03
HC	12.1 (4.9)	3–19			

*F*, *F*-test, *ƞ*^2^, eta-squared, *p < 0.05, **p < 0.01, ***p < 0.001.

FACT-Cog, The Functional Assessment of Cancer Therapy-Cognitive Function; CES-D, Center for Epidemiologic Studies Depression Scale; PROMIS, Patient-Reported Outcomes Measurement Information System Bank v1.0–anxiety; PSS, Perceived Stress Scale.

Scores on the Center for Epidemiologic Studies Depression Scale (CES-D) indicated BC (11.9 ± 7.7) reported increased symptoms of depression relative to HC (6.1 ± 1.9), *F*(1,34) = 5.4, *p* = 0.03, partial *ƞ*^*2*^ = 0.1 (Table 3; Fig. 1). The results suggest that BC reported increased symptoms of depression compared to HC on the CES-D. Scores on the Patient-Reported Outcomes Measurement Information System (PROMIS) Bank v1.0–anxiety indicated that there was no significant difference between BC (50.8 ± 7.7) and HC (47.8 ± 6.4), *F*(1,34) = 1.4, *p* = 0.3, partial *ƞ*^2^ = 0.04 (Table 3).

Scores on the Perceived Stress Scale (PSS) for BC (13.7 ± 4.9) were not statistically significant from HC (12.1 ± 4.9), *F*(1,34) = 0.9, *p* = 0.4, partial *ƞ*^2^ = 0.03 (Table 3). The results suggest that there was no significant difference between anxiety or stress levels between BC and HC groups when anxiety was measured by the PROMIS PROMIS) Bank v1.0–anxiety scale and perceived stress was measured using the PSS scale.

The statistically significant differences identified between BC and HC on FACT-Cog total and CES-D warranted further investigation on their relationship with the microbiome. Age was also included in further analysis as a potential covariate previously identified in the literature^21^. The distribution of age, FACT-Cog total, and CES-D for the two groups can be viewed in Fig. 1.

### Microbiome

**Figure 2:**
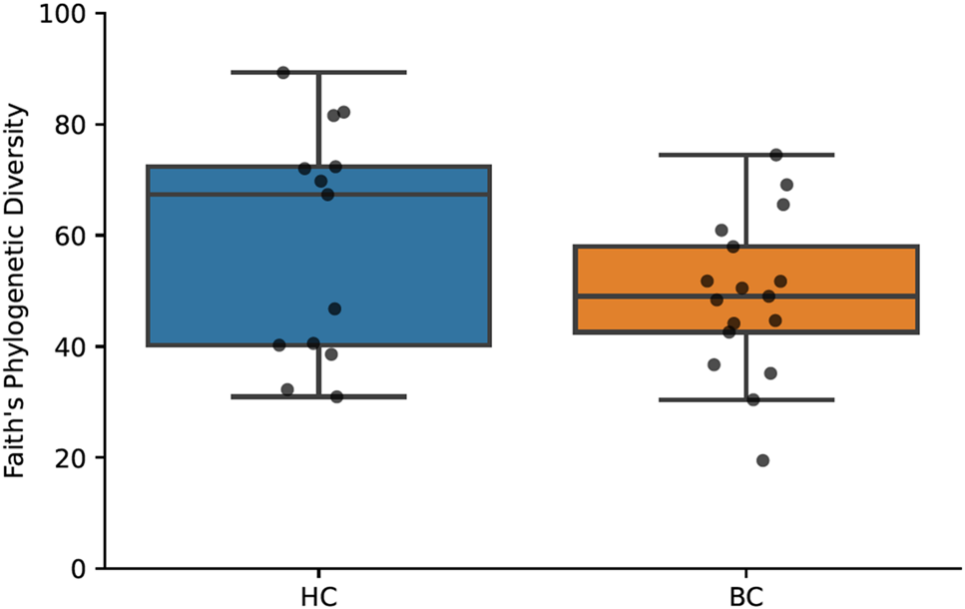
Boxplot shows Faith’s phylogenetic diversity for women who were diagnosed with and undergoing chemotherapy treatment for breast cancer (BC) versus healthy controls (HC). Boxes represent the first and third quartiles, and horizontal lines within boxes represent median values. Whiskers represent non-outlier high and low values (defined by having a value less than 1.5 times the interquartile range of the first or third quartile). Dots represent the alpha diversity values of each sample.

**Figure 3:**
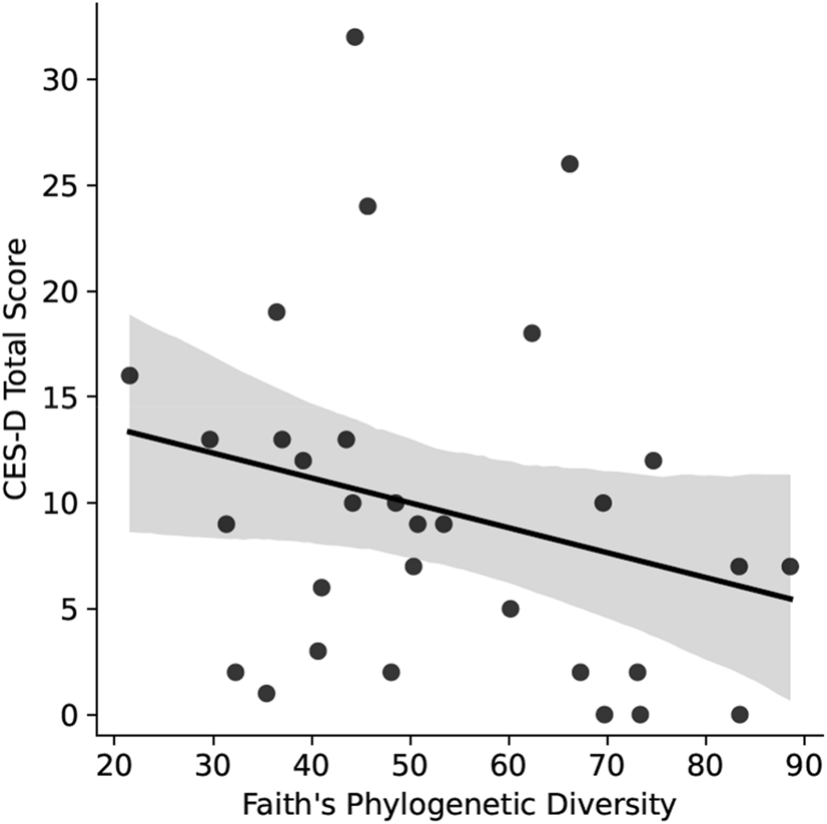
A scatterplot with a fitted linear model shows Center for Epidemiological Studies Depression Scale (CES-D) total score as a function of participants’ microbiome’s Faith’s phylogenetic diversity. Each dot represents the participant’s CES-D score in relation to the alpha diversity of the participant’s fecal microbiome sample. A high CES-D score indicates higher depression symptom severity. The solid line represents the line of best fit, and the shaded area represents a 95% confidence interval.

There was no statistically significant difference in alpha diversity as measured by Faith’s phylogenetic diversity between the BC (mean 49.5, 95% CI [42.7–56.2]) and HC (mean 57.4, 95% CI [46.3–68.6]) groups (*H*(1) = 1.2, *p* = 0.3). Distribution of the data between groups can be seen in Fig. 2. There was no significant correlation between alpha diversity and time difference between last chemotherapy treatment and stool sample collection (*p* = 0.1), FACT-Cog total (*p* = 0.1), PROMIS (*p* = 0.1), and PSS (*p* = 0.3). The correlation between alpha diversity and CES-D approached statistical significance, *r*_*s*_(28) = − 0.3, *p* = 0.07 (Fig. 3).

**Figure 4:**
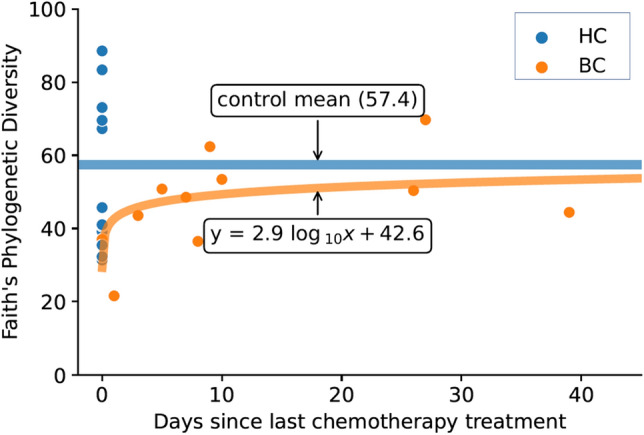
Plot shows Faith’s phylogenetic diversity as a function of time since the last chemotherapy treatment for women who were diagnosed with and undergoing chemotherapy treatment for breast cancer (BC) relative to values for participants in the healthy control (HC) group. Orange dots represent BC samples, and the orange line shows a log curve fitted to the BC data with the resulting equation *y* = 2.9 log(*x*) + 42.6. Blue dots represent samples from HC participants who did not undergo chemotherapy and were artificially given an x value of 0, and the blue line shows the mean Faith’s phylogenetic diversity for the HC group.

Figure 4 shows Faith’s phylogenetic diversity as a function of time since the last chemotherapy treatment. Among BC participants, analysis reveals a log curve restoration of the microbiome diversity after chemotherapy as best described by the equation *y* = 2.9 log(*days since last chemotherapy treatment*) + 42.6.

**Figure 5:**
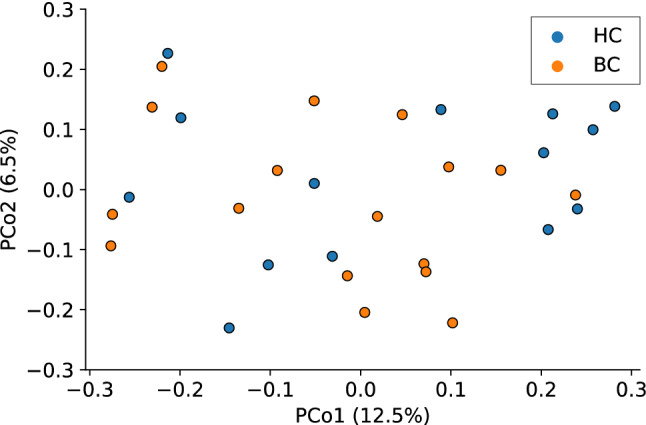
An unweighted UniFrac principal coordinates analysis shows an ordination of microbiome data, colored by chemotherapy group. Each dot represents the fecal microbiome of one participant’s sample. Orange dots indicate samples from women who were diagnosed with and undergoing chemotherapy treatment for breast cancer (BC), whereas blue dots represent samples from healthy control (HC) participants. PCo1 accounts for 12.5% of the observed variation, while PCo2 accounts for 6.5% of observed variation.

**Figure 6:**
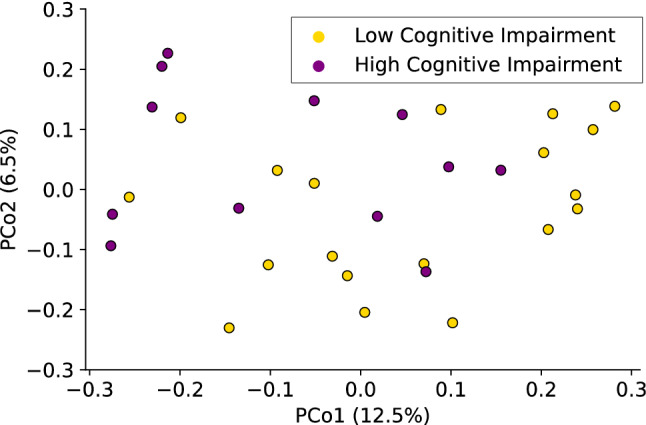
An unweighted UniFrac principal coordinates analysis shows an ordination of microbiome data, colored by self-reported FACT-Cog subscale *perceived cognitive impairments* status. Each dot represents the fecal microbiome of one participant’s sample. Purple dots indicate participants with high perceived cognitive impairment (FACT-Cog subscale *perceived cognitive impairments* < 60), whereas yellow dots represent participants with low perceived cognitive impairment. PCo1 accounts for 12.5% of the observed variation, while PCo2 accounts for 6.5% of observed variation.

There was no statistically significant difference in gut microbiome community composition between BC and HC groups as measured by Unweighted UniFrac (*pseudo-F*(30) = 1.1, *p* = 0.2) (Fig. 5), or Weighted UniFrac (*pseudo-F*(30) = 0.8, *p* = 0.5). There was a significant difference in the gut microbiome community composition between participants with high perceived cognitive impairment and low perceived cognitive impairment in the FACT-Cog subscale, *perceived cognitive impairments* as measured by Unweighted UniFrac (*pseudo-F*(30) = 1.5, *p* = 0.01) as seen in Fig. 6.

**Figure 7:**
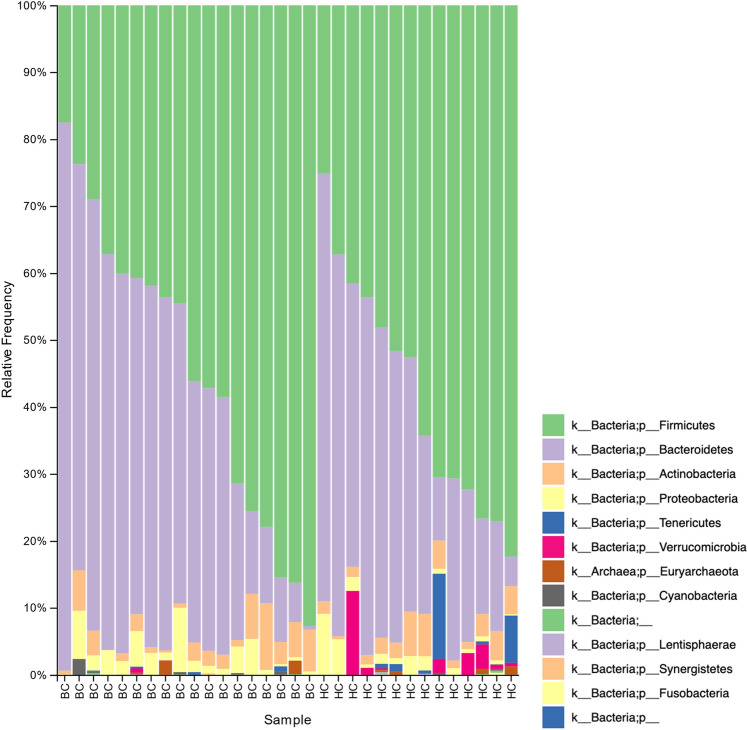
Stacked bar chart shows relative abundances of bacteria at the phylum level in the fecal microbiome of women who were diagnosed with and undergoing chemotherapy treatment for breast cancer (BC) versus healthy controls (HC). From top to bottom, stacked bars for each phylum are in order of decreasing average relative abundance and follow the order listed in the legend. k, kingdom; p, phylum; BC, women who were diagnosed with and undergoing chemotherapy treatment for breast cancer; HC, healthy controls.

**Figure 8:**
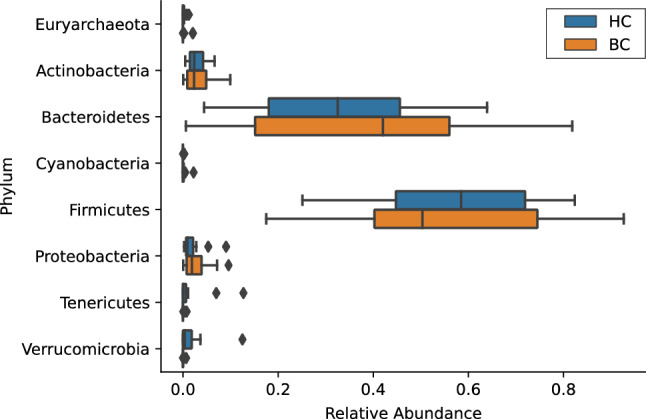
A boxplot shows the relative abundances of each phylum making up greater than 0.1% of the sequences recovered from the fecal microbiome. Boxes represent the first and third quartiles, and vertical lines within boxes represent median values. Whiskers represent non-outlier high and low values, and diamonds represent outliers as defined by having a value greater than 1.5 times the interquartile range (IQR) of the first or third quartiles. Orange boxes represent data from women who were diagnosed with and undergoing chemotherapy treatment for breast cancer (BC), whereas blue boxes represent data from healthy controls (HC).

**Figure 9:**
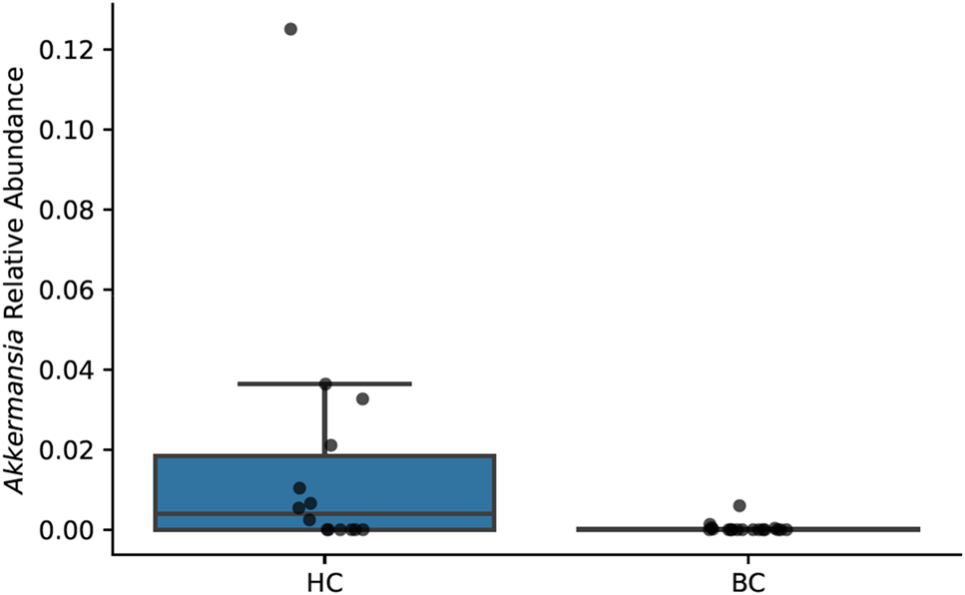
A boxplot shows relative abundance of the phylum *Akkermansia* in women who were diagnosed with and undergoing chemotherapy treatment for breast cancer (BC) versus healthy controls (HC). Boxes represent the first and third quartiles, and horizontal lines within boxes represent median values. Whiskers represent non-outlier high and low values (defined by having a value less than 1.5 times the interquartile range of the first or third quartiles). Dots represent the relative abundances of *Akkermansia* in each sample.

**Figure 10:**
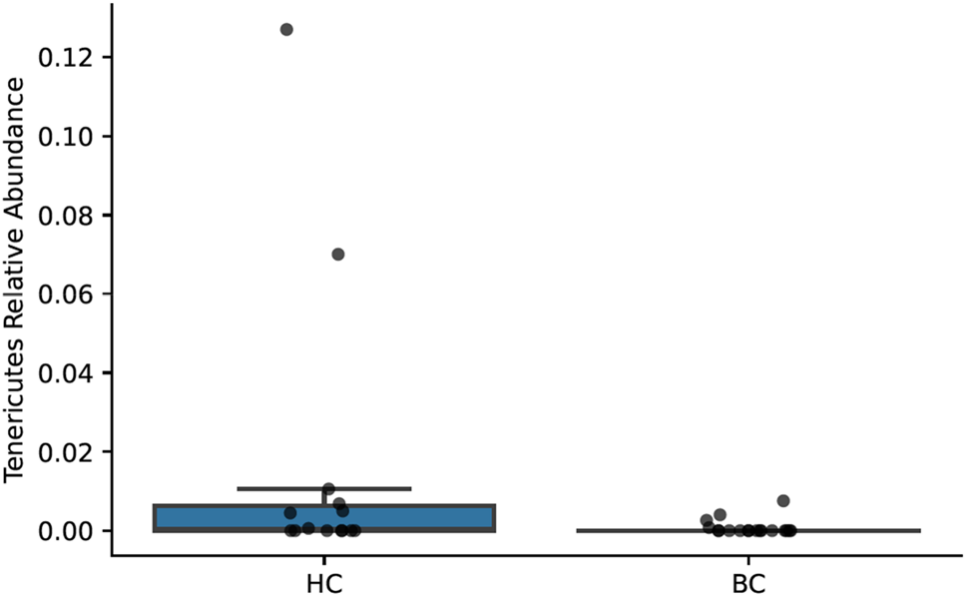
A boxplot shows the relative abundances of the phylum Tenericutes women who were diagnosed with and undergoing chemotherapy treatment for breast cancer (BC) and healthy controls (HC). Boxes represent the first and third quartiles, and horizontal lines within boxes represent median values. Whiskers represent non-outlier high and low values (defined by having a distance to the first or third quartiles less than 1.5 times the interquartile range). Dots represent the relative abundances of Tenericutes in each sample.

**Figure 11:**
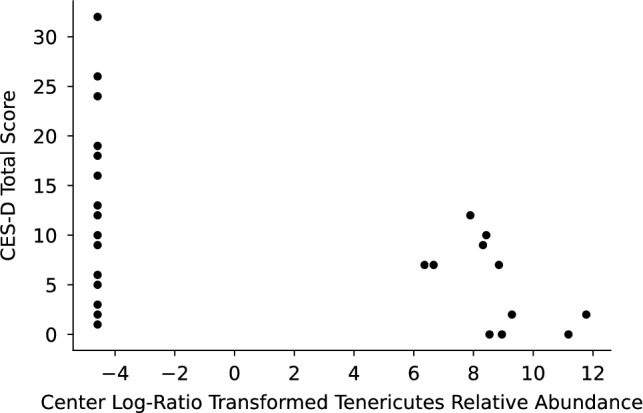
A scatterplot shows center log-ratio-transformed relative abundances of the phylum Tenericutes plotted against Center for Epidemiological Studies Depression Scale (CES-D) total score. Each dot represents the relative abundance of the phylum Tenericutes in one sample and the corresponding participant’s total CES-D score. A Spearman correlation test revealed a negative correlation between Tenericutes relative abundance and CES-D score (*r* = − 0.5, *p* = 0.002), indicating that a higher relative abundance of Tenericutes was associated with lower severity of depressive symptoms.

The phylum Verrucomicrobia had a lower relative abundance in BC samples (mean 0.0004, 95% CI [0–0.0012]) compared to HC samples (mean 0.02, 95% CI [0–0.03]; Kruskal–Wallis *H*(1) = 5.5, *p* = 0.02; Figs. 7 and 8). Of the total Verrucomicrobia reads, 99.8% were mapped to the genus *Akkermansia*, which had a lower relative abundance in BC samples (Kruskal–Wallis *H*(1) = 5.5, *p* = 0.02; Fig. 9). The phylum Tenericutes had a lower relative abundance in BC samples (mean 0.0008, 95% CI [0–0.002]) compared to HC samples that approached statistical significance (mean 0.02, 95% CI [0–0.04]; Kruskal–Wallis *H*(1) = 3.6, *p* = 0.06; Figs. 7, 8, and 10). There was a significant correlation between relative abundance of Tenericutes and CES-D total score *r*_*s*_(30) = − 0.5, *p* = 0.002 (Fig. 11), indicating that a higher relative abundance of Tenericutes was associated with lower severity of depression symptoms.

**Figure 12:**
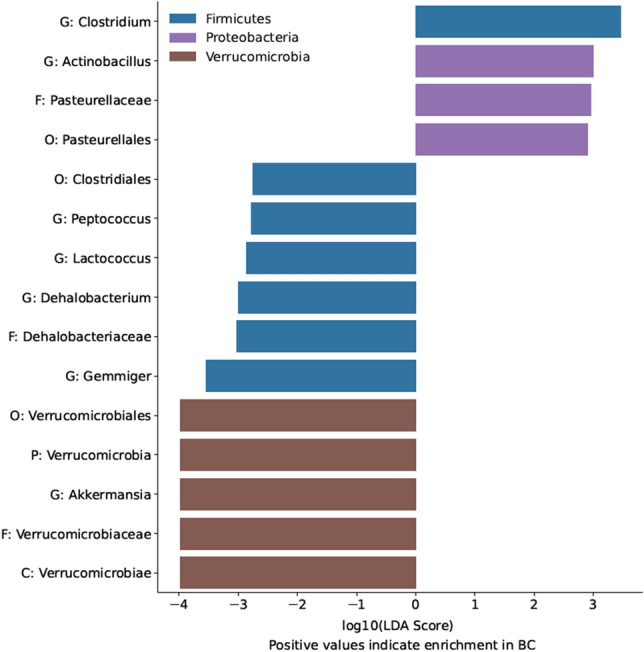
Linear discriminant analysis Effect Size **(**LEfSe) scores for taxa identified to be enriched in women who were diagnosed with and undergoing chemotherapy treatment for breast cancer (BC) relative to healthy control (HC) participants. LEfSe scores are given as the logarithm (base 10) of their importance score for linear discriminant analysis (LDA). Higher absolute values of the LEfSe scores indicate stronger enrichment in their respective groups. Positive values on the x axis indicate enrichment in the BC participants, whereas negative values indicate enrichment in the HC participants. Color represents the phylum of each taxon identified. P, phylum; C, class; O, Order; F, Family; G, genus.

Linear discriminant analysis Effect Size (LEfSe) analysis found multiple taxa differentially abundant in BC participants relative to HC. Notably, LEfSe analysis revealed lower relative abundance of Verrucomicrobia at multiple taxonomic levels (including the genus *Akkermansia*) in the BC group relative to HC group. Conversely, LEfSe analysis found enrichment of the genus *Clostridium* and multiple taxonomic levels of the order Pasteurellales, including the genus *Actinobacillus*, in the BC group relative to the HC group. Figure 12 shows the LEfSe analysis results for taxa identified to be enriched or reduced in BC relative to HC participants.

**Figure 13:**
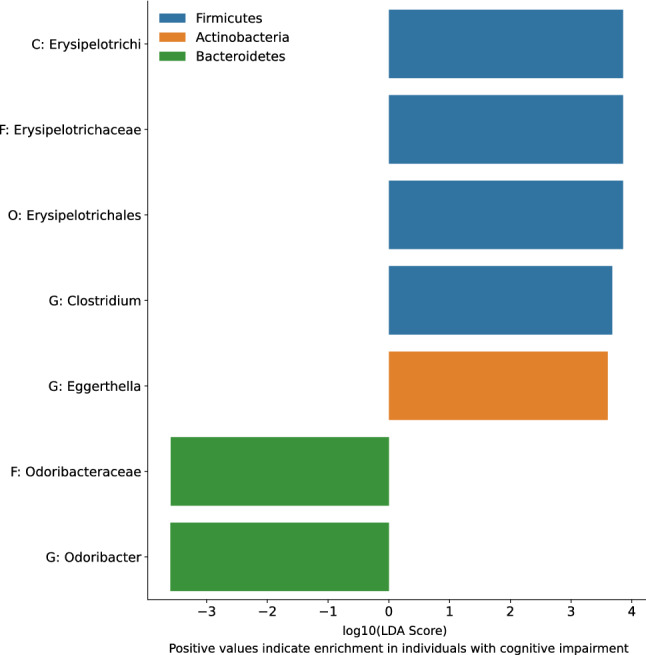
Linear discriminant analysis Effect Size (LEfSe) scores for taxa identified to be enriched in individuals with high *perceived cognitive impairments* scores (FACT-Cog < 60) relative to participants with low cognitive impairment as assessed by FACT-Cog *perceived cognitive impairments* subscales. LEfSe Scores are given as the logarithm (base 10) of their importance score for linear discriminant analysis (LDA). Higher absolute values of the LEfSe scores indicate stronger enrichment in their respective groups. Positive values on the x axis indicate enrichment in participants with high cognitive impairment scores, whereas negative values indicate enrichment in participants with low cognitive impairment scores. Color represents the phylum of each taxon identified. C, class; O, Order; F, family; G, genus.

LEfSe analysis also found multiple taxa differentially abundant in participants with high FACT-Cog *perceived cognitive impairments* scores, relative to participants with low FACT-Cog *perceived cognitive impairments* scores for both participating groups. Specifically, LEfSe analysis revealed that individuals with reported greater cognitive impairment scores (FACT-Cog < 60) had lower relative abundances of the genus *Odoribacter* and its family Odoribacteraceae; conversely, individuals with greater cognitive impairment had enrichment of the class Erysipelotrichi, as well as its family Erysipelotrichaceae and order Erysipelotrichales. Individuals with greater cognitive impairment also had an enrichment in the relative abundance of the genus *Clostridium*. Figure 13 shows the LEfSe analysis results.

### Associations of group, mood, and microbiome

Multiple hierarchical regressions explored the association between different variables with statistically significant differences between BC and HC including FACT-Cog total, CES-D, relative abundance of Verrucomicrobia, and relative abundance of Tenericutes. Age was included as a covariate in the model based on recommendation from previous literature^22,23^.

A hierarchical multiple regression allowed for the exploration of the association between group association, age, cognitive functioning, depressive symptoms, and microbiome composition. Table 4 displays a hierarchical multiple regression to determine if the addition of center log-ratio transformed Tenericutes relative abundance and FACT-Cog total scores improved the prediction of depression over and above group association and age. The full model of group association, age, center log-ratio transformed Tenericutes relative abundance, and FACT-Cog total scores to predict CES-D was statistically significant *R*^*2*^ = 0.4, *F*(4,27) = 3.9*, p* = 0.01; adjusted *R*^*2*^ = 0.4. The addition of center log-ratio transformed Tenericutes relative abundance to the prediction of CES-D (block 2) led to a statistically significant increase *R*^*2*^ = 0.1, *F*_*change*_(1,28) = 4.6*, p* = 0.01. The addition of FACT-Cog total did not lead to a statistically significant increase *R*^*2*^ = 0.04, *F*_*change*_(1,27) = 1.6*, p* = 0.2.

**Table 4:** Hierarchical multiple linear regression analysis with the predicted variable as depression as measured by CES-D total score.

Variable	Block 1			Block 2			Block 3		
	*B*	*SE B*	*β*	*B*	*SE B*	*β*	*B*	*SE B*	*β*
Group	− 8.3	2.8	− 0.01**	− 6.1	2.9	− 0.4*	− 4.0	3.3	− 0.3
Age	− 0.1	0.1	− 0.2	− 0.1	0.1	− 0.2	− 0.1	0.1	− 0.2
Tenericutes				− 0.6	0.3	− 0.3*	− 0.6	0.3	− 0.3
FACT-Cog							− 0.1	0.1	− 0.3
*R*^2^	0.2			0.3			0.4		
*F* for changes in *R*^2^	4.3*			4.6**			3.9**		

Tenericutes relative abundance was analyzed utilizing a center log-ratio transformation. *F* for changes in *R*^*2*^ *F* test for *R*-squared changes in model, *p < 0.05, **p < 0.01.

*β*, beta power; *B*, unstandardized beta coefficient; FACT-Cog The Functional Assessment of Cancer Therapy-Cognitive Function; *SE B*, standardized beta coefficient; *R*^*2*^_,_ *R*-squared.

The addition of CES-D and center log-ratio transformed Verrucomicrobia relative abundance improved the prediction of cognitive impairment measured by FACT-Cog total. The full model of group association, age, CES-D, and center log-ratio transformed Verrucomicrobia relative abundance to predict FACT-Cog total was statistically significant *R*^*2*^ = 0.5, *F*(4,27) = 5.7*, p* = 0.002; adjusted *R*^*2*^ = 0.4. The addition of CES-D did not lead to a statistically significant increase *R*^*2*^ = 0.05, *F*_*change*_(1,28) = 2.3*, p* = 0.1. The addition of center log-ratio transformed Verrucomicrobia relative abundance to the prediction of FACT-Cog total (block 3) did not lead to a statistically significant increase *R*^*2*^ = 0.001, *F*_*change*_(1,27) = 0.04*, p* = 0.8. The results are displayed in Table 5.

**Table 5:** Hierarchical multiple linear regression analysis with the predicted variable as cognitive impairment as measured by FACT-Cog total score.

Variable	Block 1			Block 2			Block 3		
	*B*	*SE B*	*β*	*B*	*SE B*	*β*	*B*	*SE B*	*β*
Group	25.9	6.9	0.6***	20.3	7.7	0.5*	19.4	9.0	0.4*
Age	− 0.2	0.2	− 0.1	− 0.2	0.2	− 0.2	− 0.3	0.3	− 0.2
CES-D				− 0.7	0.4	− 0.2	− 0.7	0.5	− 0.2
Verrucomicrobia							0.2	0.8	0.03
*R*^2^	0.4			0.5			0.5		
*F* for changes in *R*^2^	10.1***			7.8***			5.7**		

Verrucomicrobia relative abundance was analyzed utilizing a center log-ratio transformation. *F* for changes in *R*^*2*^ *F* test for *R*-squared changes in model, *p < 0.05, **p < 0.01, ***p < 0.001.

*β*, beta power; *B*, unstandardized beta coefficient; CES-D Center for Epidemiologic Studies Depression Scale; *R*^*2*^_,_ *R*-squared; *SE B*, standardized beta coefficient.

Table 6 displays a hierarchical multiple regression to determine if the addition of center log-ratio transformed Verrucomicrobia and CES-D improved the prediction of center log-ratio transformed Tenericutes over and above group association and age. The full model of group association, age, center log-ratio transformed Verrucomicrobia and CES-D to predict center log-ratio transformed Tenericutes was statistically significant *R*^*2*^ = 0.3, *F*(4,27) = 2.8*, p* = 0.05; adjusted *R*^*2*^ = 0.2. The addition of center log-ratio transformed Verrucomicrobia relative abundance to the prediction of center log-ratio transformed Tenericutes (block 2) did not lead to a statistically significant increase *R*^*2*^ = 0.04, *F*_*change*_(1,28) = 1.2*, p* = 0.3. The addition of CES-D total did lead to a statistically significant increase *R*^*2*^ = 0.1, *F*_*change*_(1,27) = 4.7*, p* = 0.04.

**Table 6:** Hierarchical multiple linear regression analysis with the predicted variable as center log-ratio transformed Tenericutes.

Variable	Block 1			Block 2			Block 3		
	*B*	*SE B*	*β*	*B*	*SE B*	*β*	*B*	*SE B*	*β*
Group	3.4	1.6	0.4*	2.3	1.9	0.3	0.3	1.9	0.04
Age	0.04	0.1	0.2	0.02	0.1	0.1	− 0.01	0.1	− 0.03
Verrucomicrobia				0.2	0.2	0.2	0.2	0.2	0.3
CES-D							− 0.2	0.1	− 0.4*
*R*^2^	0.1			0.2			0.3		
*F* for changes in *R*^2^	2.1			1.8			2.8*		

Verrucomicrobia relative abundance was analyzed utilizing a center log-ratio transformation. *F* for changes in *R*^2^ *F* test for *R*-squared changes in model, *p < 0.05.

β, beta power; *B*, unstandardized beta coefficient; CES-D Center for Epidemiologic Studies Depression Scale; *R*^2^_,_ *R*-squared; *SE B*, standardized beta coefficient.

A binomial logistic regression was performed to ascertain the effects of age, center log-transformed Tenericutes, center log-transformed Verrucomicrobia, FACT-Cog, and CES-D on the likelihood that participants were in the BC group. The logistic regression model was statistically significant, *χ*^*2*^(5) = 33.6, *p* < 0.001. The model explained 87.5% (Nagelkerke *R*^*2*^) of the variance in the BC group and correctly classified 87.2% of cases. Sensitivity was 85.7%, specificity was 88.9%, HC predictive value was 85.7% and BC predictive value was 88.9%. Of the five predictor variables, Verrucomicrobia was statistically significant while FACT-Cog and CES-D were approaching significance. A 1-unit increase in the center log-ratio transformed relative abundance of Verrucomicrobia was associated with a 2.1 times higher odds of a participant belonging to the HC group. BC group was associated with higher age, increased cognitive impairment, and increased depressive symptoms (Table 7).

**Table 7:** Binomial logistic regression predicting group association based on age, center log-transformed relative abundance of Tenericutes, center log transformed relative abundance of Verrucomicrobia, FACT-Cog, and CES-D.

Variable	*B*	*SE*	Wald	*df*	*p*	Odds ratio	95% CI for odds ratio	
							Lower	Upper
Age	− 0.3	0.2	3.4	1	0.07	0.8	0.6	1.0
Tenericutes	− 0.1	0.2	0.2	1	0.6	0.9	0.6	1.3
Verrucomicrobia	0.7	0.4	3.9	1	0.05*	2.1	1.0	4.2
FACT-Cog	0.2	0.1	3.1	1	0.08	1.2	1.0	1.4
CES-D	− 0.5	0.3	3.5	1	0.06	0.6	0.3	1.0
Constant	− 4.0	6.5	0.4	1	0.6	0.02		

*p < 0.05.

*B*, unstandardized beta coefficient; CES-D, Center for Epidemiologic Studies Depression Scale; *df*, degrees of freedom; FACT-Cog, The Functional Assessment of Cancer Therapy-Cognitive Function; *p*, statistical significance; *SE*, standard error.

## Discussion

The current study demonstrated that the BC group experienced greater disturbance in cognitive functioning and depression in association with differences in community structure of the gut microbiome, relative to HC. Cognitive functioning measured by FACT-Cog and its subscales was worse for BC compared to HC. The BC group reported higher scores for depressive symptoms compared to the HC group, as measured by the CES-D. Although microbiome richness (Faith’s phylogenetic diversity) was not significantly lower in BC compared to HC participants, and there were no significant differences in microbiome alpha and beta diversity between BC and HC participants, there was a significant difference in relative abundance of specific taxa between BC and HC. Specifically, BC participants had significantly lower relative abundance of *Akkermansia* compared to HC as assessed by a Kruskal–Wallis test and LEfSe. Examining cognitive functioning, depression, and microbiome factors together, group association of BC or HC was a strong predictor of presented differences. Overall, the study identified that BC participants experienced significant differences as compared to HC that are impacting their functioning on multiple dimensions.

BC reported greater cognitive impairment based on overall scores and subscales as measured by FACT-Cog when compared to HC. These results are similar to previous studies measuring perceived cognitive changes after receiving chemotherapy^8,11,24^. A longitudinal study of breast cancer patients treated with chemotherapy determined that higher depressive and anxiety symptoms at baseline were predictive of lower FACT-Cog scores indicating greater cognitive impairment^25^ signifying that breast cancer patients could be experiencing cognitive changes related to depression or anxiety^26^. There is also a bidirectional relationship between cognitive changes and mood as, “diminished ability to think, concentrate, or indecisiveness, nearly every day” is included as part of the criteria for Major Depressive Disorder^27^. It is also possible that cognitive impairment, depression, and anxiety share biological mechanisms, such as increased neuroinflammation^14^.

Reports on the CES-D indicated that the BC group reported higher depressive symptom scores than the HC group, suggesting BC experienced increased symptoms of depression compared to HC participants. Increases in symptoms of depression for people being treated with chemotherapy is well supported in the literature^15,16,18–20^ and could be related to a number of factors. For example, a cancer diagnosis is associated with many stressors such as fear of death, interruption of life plans, changes in body image and self-esteem, and changes in social role and lifestyle^28^. These changes along with the biological changes could contribute to the increased reported depressive symptoms.

Contrary to our hypothesis, microbiome richness was not significantly lower in BC compared to HC participants. However, a logarithmic curve fit to the data showed that richness was lowest in BC participants who recently received chemotherapy treatment and highest in BC participants who had not received chemotherapy treatment recently; as the time between the participants’ last chemotherapy treatment and fecal sample collection increased, richness tended to also increase. This supports established literature denoting that microbiome richness decreases following perturbations such as chemotherapy^17,29^ and antibiotics^30^ and returns toward the baseline after time. A recent study^31^ reported that cancer survivors less than 6 months since their last treatment had lower richness than both cancer survivors more than 6 months since their last treatment, and healthy controls. Unfortunately our study did not assess duration or frequency of chemotherapy treatment, which could have ramifications for whether or not the microbial communities could fully recover between treatments, given that repeated and frequent perturbations increase the risk of incomplete community recovery^30^. Moreover, neither study incorporated baseline measurements of richness before starting chemotherapy treatment, so it is not possible to determine whether the observations represent a post-treatment decrease in richness. In addition to the findings above, Deleemans et al.^31^ found that microbiome richness was negatively associated with depressive and cognitive symptoms. To note, individuals receiving frequent chemotherapy over a long period of time may be most susceptible to incomplete microbiome recovery and thus higher systemic inflammation. This, along with increased mental burden from treatment, could contribute to increased risk for cognitive impairment and depression symptoms.

Though there was no significant difference in microbiome beta diversity as a measure of overall community composition between BC and HC participants, we did find a relationship between community composition and cognitive impairment. Previous studies found compositional differences based on cognitive impairment status in type 2 diabetes mellitus patients^32^, individuals with and without dementia^33^, and mid-life individuals^34^. In our study, this difference in microbiome composition was characterized by a decreased relative abundance of the genus *Odoribacter* as well as increased relative abundances of the genera *Clostridium* and *Eggerthella* and the class Erysipelotrichi in BC relative to HC. The genus *Clostridium* contains the pathogen *C. difficile* and infection with *C. difficile* is associated with increased rates of delirium and decreased cognitive function in elderly patients^35^. Also, Erysipelotrichi has been consistently demonstrated to be associated with intestinal inflammation^36^, activation of inflammatory^37^ pathways, and colorectal cancer^38^. *Odoribacter*, a producer of the anti-inflammatory short-chain fatty acid butyrate, has been shown to exhibit anti-inflammatory responses in gut mucosal cell lines^39^; the relationship between decreased relative abundance of *Odoribacter* and increased cognitive impairment could be mediated by increased systemic inflammation. Overall, there are differences in microbiome composition in individuals with high perceived cognitive impairments. These were characterized by increased relative abundances of taxa that include strains with inflammatory properties and decreased relative abundances of taxa that include strains with anti-inflammatory properties, suggesting a potential mediator of systemic inflammation between the gut microbiome and cognitive impairment.

BC participants had a significantly lower relative abundance of *Akkermansia* compared to HC. Notably, the microbiome modulates inflammation following dysregulation of the intestinal epithelial barrier, and microbes such as *Akkermansia* that live on the mucosal surface are paramount in this process^40–42^. The relative abundance of *Akkermansia* is inversely related to the presence of severe conditions including irritable bowel syndrome, appendicitis, and diabetes^41^. Low relative abundance of *Akkermansia* signifies a disruption in the epithelial barrier, which in turn impacts the immune response via increased intestinal permeability^42^. For the current sample, it is hypothesized that those participants with significantly lower relative abundances of *Akkermansia* could be experiencing inflammation characterized by dysbiosis and experiencing “leaky gut”, leading to increased inflammation, physiological responses that are linked to stress responsiveness, emotional behavior, and pain modulation^18^.

In the present study the hierarchical multiple regression analyses indicated that group association was a strong predictor in each model. Therefore, a logistic regression of group association examined the variance explained by the current variables. To the research team’s knowledge there are few studies that have simultaneously examined and compared cognitive, depression, and anxiety symptom severity in concordance with microbiome alterations in BC and HC. What the current research identified is that there are multiple factors that explain the current presentation of BC participants. The current variables explained 87.5% (Nagelkerke *R*^*2*^) of the variance in group association; however, this prompts the question of what other variables can be examined to provide a fuller picture of what the BC group may experience while receiving treatment.

Overall, BC participants experienced significant changes that impacted their well-being on multiple dimensions. Additional explanations could include the distress associated with a cancer diagnosis and subsequent treatment, changes in relationships, lifestyle changes induced by cancer, lack of psychological support, and physiological changes related to a cancer diagnosis and treatment. The directionality of the presented differences was not established in the present study. However, previous research in mice has demonstrated that following exposures to psychosocial stress and treatment with antibiotics, the mice display an adrenal hormone-driven immune suppression and a concurrent deterioration in epithelial barrier functions leading to increased inflammation^43^. Increased systemic inflammation can cause increased stress reactions, and impaired cognitive functioning, and higher rates of depression^16,18,44^. The research presented here showed that BC participants experienced increased cognitive impairment, more symptoms of depression, and differences in gut microbiome community structure related to inflammation as compared to HC.

### Limitations

There are several limitations to the present study. The cross-sectional design limited our ability to make causal inferences; future studies using longitudinal designs are required to examine within-person changes in cognitive function, depression symptoms, and gut microbiome diversity and community composition in BC. In addition, cognitive functioning comprises multiple cognitive domains, such as attention, executive functions, language, memory, perception, and visuoconstruction^45^; we acknowledge that there are limitations of self-reported cognitive function, relative to cognitive tests that assess specific domains of cognitive functioning. In addition, there was considerable variability in BC assessment based on stage of chemotherapy, the type of chemotherapy received, menopausal status, and time between the assessment and the stool sample collection. There was limited variability in the sample demographics including predominately highly educated women who identified as White Non-Hispanic. With the microbiome collection, limitations included limited knowledge of participants’ diet and lifestyle factors that could contribute to alpha diversity, utilizing relative abundances limiting the awareness of total biomass, and controlling for Proteobacteria bloom through de-blooming. Another limitation included the shipping of the samples and future research should consider collecting samples in a more controlled environment. Finally, we used a cut-off for antibiotic treatments of 2 weeks, whereas some groups recommend exclusion periods of up to 6 months since last antibiotic use^46^. We could not use a more stringent antibiotic use cut-off due to high occurrence of antibiotic use in the clinic population from our community.

## Conclusions and future directions

This is one of a few studies examining the association between self-reported cognitive deficits, depression and anxiety symptoms, and gut dysbiosis in BC compared to a HC sample. The current study suggests an important relationship between these variables that warrants further longitudinal investigation to examine causal associations and the impacts of breast cancer and its treatment on multiple dimensions of a person’s well-being. The present research is important because declines in cognitive functioning and increases in depressive symptoms can impact psychological, social, physical, and occupational functioning, and depression can impact the number of side effects experienced, pain, and acceptance and adherence to chemotherapy treatment of patients^4^. Future research should address current limitations, explore mechanistic roles of the microbiome^47^, explore other factors to explain variance of distress, and explore potential interventions. The present study identified a relationship between cognitive functioning, depression, and differences in community structure of the gut microbiome. Future studies should examineg potential microbiome interventions including the use of prebiotics, probiotics, or dietary interventions to mitigate side effects^48^. Additional clinical implications include the development and testing of behavioral health interventions to help manage the distress associated with a cancer diagnosis and subsequent treatment, as well as health behavior change interventions aimed at mitigating distress and supporting the gut microbiome.

## Methods

### Participants and procedures

This cross-sectional study was approved by the Colorado Multiple Institutional Review Board (COMIRB, protocol number 16-2138) which included obtaining informed consent from all participants and adherence to all relevant guidelines and procedures. The participants consisted of two groups of women, those who were diagnosed with and undergoing chemotherapy treatment for breast cancer (BC) stage I–III, and healthy controls (HC). Additional inclusion criteria for both groups included provision to sign and date consent form, ages between 18 to 75 years, and able to read and understand English. Exclusion criteria for both groups were sensory or speech/language deficits, treatment with antibiotics in the previous 14 days, other comorbid diseases including congestive heart failure, hepatic or renal disease, chronic obstructive pulmonary disease, autoimmune disorders, and coeliac disease. For the BC group, those with stage IV breast cancer were excluded due to the disease having metastasized and, as a result, they receive very different treatment protocols, and thus were not included in this study. BC participants' cancer stage and menopausal status were defined using American Joint Commission on Cancer version 7 (AJCC v 7) definition^49^. The BC group was recruited from the Anschutz Medical Campus Breast Clinic, and the HC group consisted of a community sample that was recruited in the Denver, Colorado area via informational flyers and referrals made by participating breast cancer patients.

**Figure 14:**
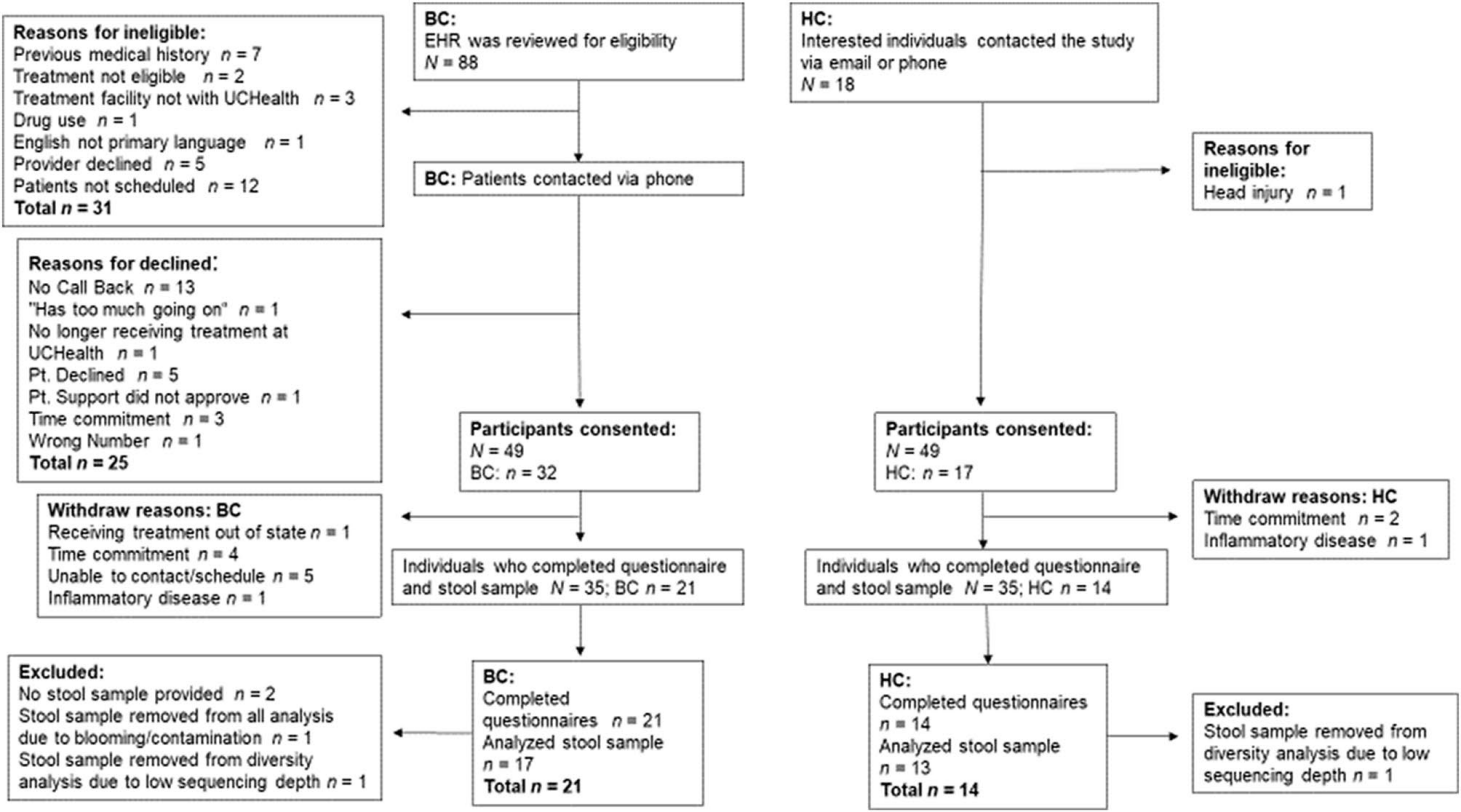
Consort diagram depicts the recruitment of women who were diagnosed with and undergoing chemotherapy treatment for breast cancer (BC) and healthy control (HC) groups.; EHR, Electronic Health Record; UCHealth, University of Colorado Health; pt., patient.

Recruitment of study participants took place between May 17, 2018, and March 23, 2020. Recruitment ended early because of the COVID-19 pandemic. A total of 106 participants were screened (88 BC and 18 HC). Of those screened, 32 BC and 17 HC consented to participate. Of the 49 participants who enrolled in the study, a total of 35 participants (*N* = 21, BC; *N* = 14, HC) participated in the study resulting in a 71% study retention rate (Fig. 14).

All participants completed a web-based survey, administered via RedCap^50,51^, that included self-reported demographic information and measures of cognitive impairment, depressive symptoms, and anxiety symptoms. In addition, participants were provided a kit for simple acquisition of a stool sample to be collected in the privacy of their homes. The stool sample was obtained by participants using a sterile swab to collect a small amount of stool from toilet paper following a bowel movement. Participants were instructed to record the date and time of fecal sample collection. The sample was shipped with an ice pack to the investigators for processing, storage, and analysis.

A total of 21 participants of the BC group completed the questionnaires and 19 participants completed the stool sample. In the HC group, 14 participants completed the questionnaires, and 13 participants completed their stool sample. Reporting of gut microbiome data in this report is consistent with the Strengthening The Organization and Reporting of Microbiome Studies (STORMS) guidelines for human microbiome research^52^.

### Cognitive functioning

The FACT-Cog^24^, is a self-report measure of cognitive problems developed specifically for use with cancer patients. The 37-item measure uses a Likert scale ranging from 0 (“not at all”) to 4 (“very much”) to assess cognitive challenges. The four subscales include *perceived cognitive impairments, comments from others* (indicating the participant’s perception of people making comments on their cognitive functioning)*, perceived cognitive abilities,* and *quality of life*. The FACT-Cog shows concurrent validity with previous measures (*r* = 0.7), and Cronbach’s α for all the FACT-Cog domain scores range from α = 0.7 to 0.9^24^. A score of less than 60 indicates that participants are reporting cognitive problems as measured by the FACT-Cog subscale *perceived cognitive impairments*^53^.

### Depression, Anxiety, and Stress

The CES-D^54^, developed at the National Institutes of Health, is widely used as a self-report measure of depressed mood. The 20-item Likert scale measure is used to identify the frequency of depressive symptomatology “in the past week or so.” The CES-D has good internal consistency in the general population^54^, and in a group of breast cancer participants (α = 0.9)^55^. A cutoff score of 16 or higher indicates individuals at risk for clinical depression^56^.

The PROMIS^57^, developed under the sponsorship of the National Institutes of Health, assesses multiple dimensions of mental health. Specifically, the PROMIS-Ca Bank v1.0–Anxiety scale used in this study assesses emotional distress-anxiety and was validated in an adult cancer population. Individual items enquired about the frequency of symptoms during the previous 7 days, ranging from “Never” to “Always.” The emotional distress-anxiety scale demonstrates reliability of *r* = 0.9 between the short form and the entire PROMIS bank^57^.

The PSS^58^, a widely used measure of the perception of stress, assesses the degree to which situations in one's life within the last month are appraised as stressful. The PSS consists of 10 items such as, “How often have you felt confident about your ability to handle your personal problems.” The answers range from 0 (“Never”) to 4 (“Very often”). In a meta-analysis of 12 studies using the PSS, Cronbach’s α > 0.7, and the validity of the measure was moderately or strongly correlated with several previously established measures^58^.

### Microbiome sample collection, DNA extraction, and sequencing

Microbiome samples were collected at home by participants using sterile double tipped swabs (Culture Swab™ EZ II System Beckton Dickinson and Company, Franklin Lakes, NJ, USA). Though the terms “microbiome” and “microbiota” are often used interchangeably, we use the term microbiome in our study, as its definition of the genomic information in the gut environment, which we analyzed through 16S rRNA gene sequencing^59^. The sample was shipped with an ice pack to the University of Colorado Boulder and frozen at − 80 °C until processing and analysis. For microbiome samples, DNA was extracted from a sterile swab using the PowerSoil DNA extraction kit (Cat. No. 12955-4, Qiagen, Valencia, CA, USA) according to the manufacturer’s instructions. Marker genes in isolated DNA were polymerase chain reaction (PCR) amplified using GoTaq Master Mix (Cat No. M5133, Promega, Madison, WI, USA); 515 F (5′-GTGCCAGCMGCCGCGGTAA-3′) and 806 R (5′-GGACTACHVGGGTWTCTAAT-3′) primer pair (Integrated DNA Technologies, Coralville, IA, USA) targeting the V4 hypervariable region of the 16S rRNA gene modified with a unique 12-base sequence identifier for each sample and the Illumina adapter, per the Earth Microbiome Project Protocol^60^. The thermal cycling program consisted of an initial step at 94 °C for 3 min followed by 35 cycles (94 °C for 45 s, 55 °C for 1 min, and 72 °C for 1.5 min), and a final extension at 72 °C for 10 min, similar to the Earth Microbiome Project Protocol^60^. PCRs were run in duplicate, and the products from the duplicate reactions were pooled and visualized on an agarose gel to ensure successful amplification. PCR products were cleaned and normalized using a SequalPrep Normalization Kit (Cat. No. A1051001, ThermoFisher, Waltham, MA, USA) following the manufacturer’s instructions. The normalized amplicon pool was sequenced on an Illumina MiSeq run by using V3 chemistry and 600 cycles, 2 × 300-bp paired-end sequencing^61^. No positive (mock community) controls were used, but PCR blank negative controls were. All library preparation was conducted by the Lowry Lab at the University of Colorado Boulder Wilderness Place facility in one batch, and sequencing was conducted in one batch at the University of Colorado Boulder BioFrontiers Next-Gen Sequencing core facility.

### Microbiome processing

Microbiome data were processed using Python-based packages (Python 3.6.12) and QIIME 2 2019.4 (denoising and taxonomy assignment). To identify reads corresponding to each sample, multiplexed sequencing data were demultiplexed using the q2-demux plugin, with reverse-complement barcode parameters passed, resulting in a median of 204,548 forward reads and 204,548 reverse reads per sample. Demultiplexed paired-end sequences were denoised using DADA2, for which forward reads were truncated at 295 base pairs, and reverse reads were truncated at 237 base pairs, resulting in a median of 36,501 reads per sample with a total of 2864 unique suboperational-OTUs (sOTUs). The q2-fragment-insertion plugin was used to create a phylogenetic tree via SATé-enabled phylogenetic placement (SEPP) and to filter outlier reads. Based on Amir et al., facultative anaerobes known to bloom during sample shipping were removed^62^. Taxonomy was assigned using a naïve-Bayes classifier based on the latest Greengenes 16S rRNA gene database available as of August 2019 via the QIIME 2 interface (gg_13_8), and 811 chloroplast and mitochondrial reads were filtered from the dataset. All abundances were assessed in terms of relative abundance, such that the sum for all samples added to 1. One remaining sample, which contained over 60% relative abundance of Proteobacteria belonging to the family Comamonadaceae, was removed from all microbiome analyses due to likely contamination or blooming. Additional Python packages (SciPy.stats, Scikit-bio, versions concurrent with the QIIME 2 2019.4 environment) were used for statistical tests on QIIME 2-generated data.

Each sample was rarefied to 13,055 reads for alpha and beta diversity analyses via q2-diversity’s core-metrics-phylogenetic pipeline. This rarefaction depth excluded 1 BC sample and 1 HC sample from diversity analyses due to sequencing depth below 13,055 reads, but these samples were retained for taxonomic analysis. As such, of 19 total BC stool samples provided, after 1 sample was excluded due to blooming or contamination, 18 were analyzed for taxonomic composition, and 17 were analyzed for diversity. Of 14 total HC stool samples provided, 14 were analyzed for taxonomic composition, and 13 were analyzed for diversity. Alpha diversity was assessed by Faith’s phylogenetic diversity, a richness metric^63^. Bacterial composition (beta diversity) was analyzed using both unweighted and weighted UniFrac phylogenetic distance metrics.

### Statistical analysis

A Chi-squared goodness-of-fit test determined whether the distribution of categorical demographic variables was different between BC and HC. An ANOVA determined if there were statistically significant differences between the means of the BC and HC groups on the FACT-Cog, CES-D, PROMIS-Ca Bank v1.0–Anxiety scale, and PSS. Inspection of the boxplot indicated relatively normal distribution, which reflects the expected population scores. Normality of distribution was assessed by the Shapiro–Wilk's test. No transformations were applied to the data. Additionally, multivariate regression and logistic regression models tested different models involving group membership, age, FACT-Cog total score, CES-D total score, and the relative abundances of the phyla Tenericutes and Verrucomicrobia analyzed utilizing a center log-ratio transformation.

The effects of diagnosis and chemotherapy treatment for breast cancer were assessed using analysis of cross-sectional differences between BC and HC groups. Differences in alpha diversity between groups were determined by Kruskal–Wallis non-parametric rank test. Differences in beta diversity between groups were assessed via permutational multivariate analysis of variance (PERMANOVA). Comparisons of relative abundances of microbial taxa between groups were performed using Linear Discriminant Analysis using LDA Effect Size (LEfSe) with the Segata et al. online interface^64^. Taxa with an LDA value of 2.0 or greater and a two-tailed *p* value ≤ 0.05 with Kruskal–Wallis and pairwise Wilcoxon analyses were considered significantly enriched. For testing on specific taxa, a center log-ratio transformation was performed to address normality. All statistical tests performed were two-tailed with an alpha of 0.05, if applicable.

## Data Availability

Source code for analysis can be found online at https://github.com/sterrettJD/Chemobrain. Raw sequencing data and sample metadata can be found at qiita.ucsd.edu using the study identifier 14669 (https://qiita.ucsd.edu/study/description/14669) or at the European Nucleotide Archive using the identifier ERP139431 (https://www.ebi.ac.uk/ena/browser/view/PRJEB54599).

## Abbreviations

ANOVA: Analysis of variance
BC: Women who were diagnosed with and undergoing chemotherapy treatment for breast cancer
CES-D: Center for Epidemiologic Studies Depression Scale
CNS: Central nervous system
EHR: Electronic Health Record
FACT-Cog: Functional Assessment of Cancer Therapy-Cognitive Function
HC: Healthy controls
IL: Interleukin
LDA: Linear discriminant analysis
LEfSe: Linear discriminant analysis effect size
NFκB: Nuclear factor kappa B
PCR: Polymerase chain reaction
PERMANOVA: Permutational multivariate analysis of variance
PROMIS: Patient-Reported Outcomes Measurement Information System
PSS: Perceived Stress Scale
pt: Patient
SEPP: SATé-enabled phylogenetic placement
sOTUs: Sub-operational taxonomic units
UCHealth: University of Colorado Health System
